# Great Sympathetic Nerve

**Published:** 1863-10

**Authors:** 


### GREAT SYMPATHETIC NERVE.

From a recent number of the *Journal de la Physiologie de l'Homme et des Animaux*, published by Brown-Sequard, we extract the following, as a resume of the results of certain vivisections lately made by Claude Bernard, for the purpose of determining the office of the Sympathetic Nerve. It has been determined, beyond all doubt, by Bernard, that this nerve exercises an influence upon the calibre of blood-vessels, as well as upon the temperature of the parts to which it is distributed; also, that the Sympathetic Nerve exercises these functions wholly independent of the cerebro-spinal system.

"In conclusion," says he, "to make a summary of the results of my experiments upon this nerve,—they demonstrated that the nerves which are sent to the vessels, (and which he terms vascular nerves,) and those which preside over calorification, are topographically and physiologically independent from those which are known as the muscular, (that is, the common motor nerves.) From this we derive this general proposition, that the vascular circulatory apparatus possesses a special vaso-motor system, and that the movement of the blood can be accelerated or retarded in the vessels, either locally or generally, without any participation of the cerebro-spinal motor nervous system. The local and functional congestions which occur in certain organs at certain periods, are examples of circulatory movements in a physiological state. In fevers, we have presented to us another striking example, occurring in a pathological state.

"I am not able to close this communication without adding certain reflections relative to the relations which my experiments hold in reference to the ideas which generally obtain among physiologists in respect to the great Sympathetic Nerve. It should be mentioned that,during a long period, physiologists have discussed, and are still discussing, the nature of this nerve, and it has been a question whether the sympathetic system constitutes an apparatus independent of the cerebro-spinal, or whether it is dependent upon, and sustained by the latter system;—and certain physiologists seem to think that the solution of this question would be equivalent to solving the problem of this nerve. I am, then, perhaps, asked, what I deduce from my experiments in this respect;—whether I conclude from them that the vascular branches of the Sympathetic Nerve arise from the spinal marrow, or whether they are independent of it? I reply, that I believe that no person, to-day, is capable of answering that query, at least, in an absolute manner. I know very well, that, in cutting the roots of the nerves whence spring the sciatic, or those of the brachial plexus,without obtaining any calorific phenomena in the limbs, this by no means proves that the vascular and calorific branches found in the Sympathetic do not arise at some point higher or lower than where the section was made. In certain cases, it seemed that I procured calorific phenomena in the hind-leg of animals, after acting upon the marrow at some higher point; also, I have witnessed an augmentation of temperature occur in the fore-leg and ear, after cutting the sympathetic cord at a point on a level with the third and fourth dorsal pair of nerves, and this occurred without any action upon the pupil;—all this appears to prove that the calorific effects are distinct from the action upon the pupil and eye-ball. (For after section of the Sympathetic in the upper portion of the body, Petit found that there ensued contractions of the pupil, and sinking of the eye into its orbit. —*Translator.*) In that portion of the marrow which is comprised between the brachial and lumbar plexuses, as well as in other portions of the medulla spinalis, there could exist, without doubt, centres which act, either directly or indirectly, by reflex action, so as to produce the vascular and calorific effects of the great Sympathetic. * * In my opinion, the vascular and calorific nerves are motor nerves; before commingling with the nerves of mixed function, these nerves constantly emanate from the ganglia of the Sympathetic, in which points they may be considered united together as in a plexus."

Hence, then, according to the opinion of Claude Bernard, one of the great functions of the Sympathetic is to serve as an excito-motor of the blood-vessels; likewise, either directly or indirectly, it influences the heat of the part to which it is distributed.—*San Francisco Med. Press.*

